# Rat adipose-derived mesenchymal stem cells aging reduction by zinc sulfate under extremely low frequency electromagnetic field exposure is associated with increased telomerase reverse transcriptase gene expression

**Published:** 2017

**Authors:** Ezzatollah Fathi, Raheleh Farahzadi, Reza Rahbarghazi, Hossein Samadi Kafil, Rahman Yolmeh

**Affiliations:** 1 *Department of Clinical Sciences, Faculty of Veterinary Medicine, University of Tabriz, Tabriz, Iran; *; 2 *Cardiovascular Research Center, Tabriz University of Medical Sciences, Tabriz, Iran; *; 3 *Stem Cell Research Center, Tabriz University of Medical Sciences, Tabriz, Iran;*; 4 *Drug Applied Research Center, Tabriz University of Medical Sciences, Tabriz, Iran; *; 5 *Shefa Neurosciences Research Center, Khatam Al-Anbia Hospital, Tehran, Iran.*

**Keywords:** Adipose, Aging, Electromagnetic field, Mesenchymal stem cells, Zinc sulfate

## Abstract

Zinc as an essential trace element was reported to be involved in regulation of the growth and aging of cells. In this study, rat adipose-derived mesenchymal stem cells were exposed to extremely low frequency electromagnetic field (ELF-EMF) of 50 Hz and 20 mT to evaluate whether exposure to ELF-EMF in the presence of zinc sulfate (ZnSO_4_) affects the telomerase reverse transcriptase (TERT) gene expression and aging in mesenchymal stem cells (MSCs). The cell plates were divided into four groups including group I (control without ZnSO_4_ and ELF-EMF exposure); group II (ELF-EMF-exposure without ZnSO_4_); group III (ZnSO_4_ treatment without ELF-EMF exposure) and group ІV (ELF-EMF exposure with ZnSO_4_). In the presence of different concentrations of ZnSO_4_, cells viability, TERT gene expression and percentage of senescent cells were evaluated using colorimetric assay, real-time PCR and senescence-associated β-galactosidase activity assay, respectively. In this experiment, cells were exposed to ELF-EMF for 30 min per day for 21 days in the presence and absence of ZnSO_4_. The results revealed that ELF-EMF leads to a decrease in the expression of TERT gene and increase in the percentage of senescent cells. However, the ZnSO_4_ could significantly increase the TERT gene expression and decrease the aging of ELF-EMF-exposed MSCs. It seems that ZnSO_4_ may be a beneficial agent to delay aging of ELF-EMF-exposed MSCs due to the induction of TERT gene expression.

## Introduction

Everyone is exposed to a complex mix electromagnetic field (EMF) that permeates our environment.^1^ The effects of extremely low frequency electromagnetic fields (ELF-EMFs) on biological systems including anti-oxidative enzymes, cell proliferation and differentiation of stem cells have been investigated.^2^^-^^4^ Today, the hazardous or beneficial biological effects of EMF on human and animals are the subject of many studies.^3^ In recent years, some studies have shown an association between exposure to ELF-EMF and changes in the expression of genes involved in the metabolic processes, response to stress and cell proliferation, aging and death.^4^ It has been reported that EMF of 75 Hz, 2 mT increases cell proliferation rate of human mesenchymal stem cells (hMSCs).^5^ Moreover, it has been shown that EMF of 50 Hz, 1 mT can enhance cell proliferation rate and inhibit programmed cell death in human neuroblastoma and rat pituitary cells.^6^ On the contrary, it was found that EMF of 50 Hz, 20 mT can inhibit growth and metabolism of hMSCs.^4^

Reportedly, telomeres and telomerases provide a way to delay aging.^7^ Telomeres are the ends of linear chromosomes that protect the chromosomes from end-to-end fusion and maintain genomic integrity.^7^ Telomerase is a ribonucleoprotein enzyme that uses its RNA as a template for synthesizing telomeric DNA on chromosomal ends.^7^ Telomerase reverse transcriptase (TERT) is the catalytic component of telomerase enzyme considered to play an essential role in the activation of telomerase.^8^ TERT expression is tightly regulated and it is only found highly expressed in germ cells, stem cells and ∼ 90% of cancer cell lines containing a functional telomerase.^9^ There is a correlation between telomere length and ageing. On the other hand, aging is characterized by presenting cells with critically short telomeres.^10^^,^^11^ Furthermore, some studies have suggested that aging is the result of oxidative damage accumulation caused by free radicals generated as by-products during normal metabolism.^12^ Moreover, the increase in free radicals are known to induce apoptosis and aging and ELF-EMF has been shown to increase free radical levels.^13^ With these explanations, much attention has been given to the elements preventing cellular aging in the last decade.^14^ It has been shown that L-carnitine as an antioxidant can be used as a good candidate for extending the replicative life-spans of aged MSCs by increasing TERT gene expression and telomere length.^15^ Further, it has been documented that radio electric asymmetric conveyer (REAC) directly influences the expression of TERT and telomere length during aging of hMSCs by transcriptionally enhancing the expression of TERT and counteracting telomere shortening.^16^

It is believed that Zinc ion (Zn^+2^) plays a critical role in various biological processes like cell growth and cycling and has an important role in many processes that are related to the scavenging of reactive oxygen species, brain aging and onset of age-related neurodegenerative diseases.^17^^-^^19^ There are some reports that Zn^+2^ deficiency causes apoptosis in various cell and tissue types A number of studies have shown the correlation between Zn^+2^ and apoptosis and suggested that Zn^+2^ may function as an antioxidant in cells.^20^^,^^21^ One anti-apoptotic mechanism of Zn^+2^ is its ability to minimize oxidative damage to cellular organelles.^20^

The use of human beings as the test model is impossible and the long-term observation of a test group could be affected by other factors such as dietetic habit and inherited disease.^22^ Certainly, stem cells could be the best choice for experimental studies.^23^ Any small influence on stem cells may cause unexpected results to their related adult cells.^4^^,^^24^ Furthermore; studies on the influence of ELF-EMF on stem cells are rare. To date, the effects of ELF-EMF in the presence of zinc sulfate (ZnSO_4_) are yet to be reported on the TERT gene expression and senescent adipose-derived mesenchymal stem cells (ADSCs). This study was carried out to evaluate the effect of ZnSO_4_ on TERT gene expression and aging of ELF-EMF-exposed rat primary adipose MSCs. 

## Materials and Methods

All chemicals, unless specified, were purchased from Sigma-Aldrich (St. Louis, USA). All cell culture plasticware was from SPL Life Sciences (Pocheon, Korea). The type of this study was experimental study.

**Isolation and culture of ADSCs. **In this study, consent was given by the ethical committee of the University of Tabriz (Tabriz, Iran). Male rats (6 to 8 week-old) were euthanized using ketamine (87 mg kg^-1^; Alfasan, Woerden, The Netherlands) and xylazine (13 mg kg^-1^; Alfasan) and epididymal adipose tissues were collected under sterile conditions. The ADSCs were isolated from adipose tissue as previously described.^25^ Briefly, adipose tissues were transported to the laboratory in phosphate-buffered saline (PBS) solution supplemented with 2% (v/v) fetal bovine serum (FBS) within 2 hr post-operation, carefully dissected and minced using a sterile scissor. Subsequently, the fat was washed extensively with Dulbecco’s modified Eagle’s Medium (DMEM) supplemented with 5% penicillin/ streptomycin. Tissue was then enzymatically dissociated for 30 min at 37 ˚C using 0.075% (w/v) collagenase type I (Invitrogen Ltd., Paisley, UK). The solution was neutralized by addition of DMEM containing 10% (v/v) FBS and centrifuged at 800 *g* for 5 min. The cell pellet was filtered through a 75-µm nylon mesh to remove cellular debris and re-suspended in DMEM containing 10% (v/v) FBS and 1% (v/v) penicillin/streptomycin solution. Following incubation, the plates were washed extensively with PBS to remove non-adherent cells, red blood cells and remaining debris. Cultures were maintained at subconfluent levels in a 37 ^°^C incubator with 5% CO_2_ and passaged with trypsin ethylenediaminetetra acetic acid (EDTA; Invitrogen) when required.^26^^,^^27^

**Detection of ADSCs markers by Immunocytochemistry. **A total of 4 × 10^4^ cells from passage four were seeded in a 24-well culture plate. After one day of culture, cells were washed three times with PBS and fixed in 4% paraformaldehyde for 30 to 60 min at room temperature. After fixation, the paraformaldehyde was removed and cells were then washed two times with PBS and once with PBS and 1% bovine serum albumin (BSA). Cells were incubated overnight at 4 ^°^C with a 1:100 dilution of monoclonal antibodies (mAbs) against CD73, CD90, CD45 and CD56 (BD Biosciences, San José, USA) in PBS and 1% BSA. Cells were then washed three times with PBS and 1% BSA and incubated with a 1:500 dilution of biotin-conjugated mouse monoclonal IgG1 antibody against rat in PBS and 1% BSA for one hr. After three washings with PBS, a 1:500 dilution of Streptavidin Alexa Fluor™ 488 conjugate (Molecular Probes, Eugene, USA) was added for 1 hr. Cells were washed three times with PBS and nuclei were stained with 7.50 µM propidium iodide (PI) for 15 min. After washing three times with PBS, cells were covered with Vectashield mounting medium (Vector Laboratories Inc., Burlingame, USA) and visualized under the fluorescence microscope. The pluripotent capacity of the isolated MSCs was confirmed with adipogenic and osteogenic differentiation.

**Adipogenic differentiation and Oil Red-O staining. **Cells at passage four were seeded at a density of 20 × 10^3^ cells per cm^2^. Sub-confluent cells were incubated in adipogenic induction medium containing 0.50 mM 1-methyl-3 isobutylxanthine, 1 µM dexamethasone, 10 µg mL^-1^ insulin and 200 µM indomethacin; the medium was changed every 3 to 4 days. At the end of the day 21, formalin-fixed cells were washed in 50% isopropanol and stained with Oil Red-O for 15 min and lipid droplets were observed by a light microscope.^28^

**Osteogenic differentiation and alizarin red staining. **The ADSCs were plated in the same manner as described for the adipogenesis. After ADSCs reached about 90% confluency, osteogenesis was induced by osteogenic induction medium containing 10% FBS, 10 nM dexamethasone, 100 U mL^-1^ penicillin, 100 µg mL^-1^ streptomycin, 10 mM b-glycerophosphate, and 0.05 mM L-ascorbic acid-2-phosphate for 21 days. To confirm the successful osteogenic differentiation, after 21 days of culture, calcium depositions were stained with alizarin red staining. Briefly, cells were washed twice with excess PBS and fixed in a solution of 2% (v/v) formaldehyde. After 15 min, alizarin red (40 mM, pH 4.10) was added to each well. The plates were incubated at room temperature for 20 min and then, they were washed two to three times with PBS for 5 min to reduce non-specific staining.^29^

**RNA extraction and reverse transcription (RT)-PCR analysis of bone and adipose tissue-specific genes expressions. **Total RNA from the osteogenic and adipogenic differentiated cells was isolated using trizol reagent (Invitrogen).^26^ Extracted cellular RNA was dissolved in diethyl phosphorocyanidate-treated water. After DNase treatment by DNase I amplification grade kit (Invitrogen), 2 µg RNA were used for the first strand cDNA synthesis in a total volume of 20 µL according to the manufacturer’s guidelines. The thermal program for PCR was an initial denaturation for 15 min at 94 ^°^C followed by 28 cycles of 1 min at 94 ^°^C, 1 min at 55 ^°^C, 56^°^C, 59 ^°^C, 58 ^°^C and 56 ^°^C (an optimised annealing temperature for alkaline phosphatase (ALP), osteocalcin (OCN), PPAR-alpha, PPAR-gamma and GAPDH primers, respectively), and 1 min at 72 ^°^C with a final 10 min extension at 72 ^°^C. Reaction mixtures for PCR included 20 ng cDNA, 10 µL TEMPase Hot Start 2x Master Mix І Blue (Ampliqon A/S, Odense M, Denmark), 0.50 µM of each antisense and sense primer as follows: ALP (NM_ 013059.1): 5' CCTTGAAAAAT GCCCTGAAA-3' (forward), 5'- CTTGGAGAGAGCCACAAAGG -3' (reverse), osteocalcin (OCN) (NM_001278484.2): 5'- GTCCCACACAGCAACTGC-3' (forward), 5'-CCAAAGGCTGAAGCTGCCG-3' (reverse). PPAR-alpha (NM_013196.1): 5'- CCCTGCCTTCCCTGTGAAC TGAC-3' (forward), 5'-GGGACTCATCTGTACTGGTGGGGAC-3' (reverse). The PPAR-gamma (NM_013124.3): 5'-GGTGA AACTCTGGGAGATCC-3' (forward), 5'-TGAGGGAGTTTGAAGACTCTTC-3' (reverse). GAPDH (NM_017008.4): 5'-ATGACTCTACCCACGGCAAG-3' (forward), 5'-CTGGAGATGGTGATGGGTT-3' (reverse) and 9. Amplified PCR products were analyzed by ethidium bromide staining after 1.5% agarose gel electrophoresis.^30^^,^^31^ The PCR primers were designed with Oligo primer design software (version 7.0; National Biosciences, Plymouth, USA).

**Determination of suitable concentration of ZnSO**_4_ **and cell proliferation by methylthiazoletetrazolium (MTT) assay. **The tetrazolium-based colorimetric assay MTT test measures the mitochondrial activity in the cell culture, which reflects the number of viable cells. Briefly, at passages three to aux cells were trypsinized, counted with hemocytometer and seeded at a density of 4 × 10^3^ cells per well to a 96-well culture plate. Cells were incubated for 24 hr at 37 ˚C in a humidified environment with 5% CO_2_ to grow the cells in a monolayer. The ZnSO_4_ was added to the wells at final concentrations of 1.40, 0.14 and 0.014 µg mL^-1^. Control wells were prepared by addition of corresponding medium. The stock MTT dye solution (2 mg mL^-1^) was added to each well after the plates were incubated at 37 ˚C in a 5% CO_2_ incubator for 21 days. Following incubation for 4 hr, the supernatant was removed and dimethyl sulfoxide (100 µL) was added. The optical density of each well was measured in an ELISA reader at a wavelength of 570 nm. ^32^^,^^33^

**The ELF-EMF** **system and exposure protocol.** The EMF device was designed and manufactured by University of Tabriz in Iran. The EMF was generated by a parallel set of Helmholtz solenoid coils with 500 turns of 0.70 mm coated copper wire. Each solenoid diameter was 27 cm. The coil was placed into the cell incubator; the field was set to 50 Hz electromagnetic field frequency and generated a magnetic flux density of 20 mT as previously described.^4^ The EMF-exposed plates were placed in the center half way between the plains of coils to receive a uniform field for 30 min per day for 21 days. Sham-exposed control samples were kept under the same conditions but in another incubator, without using EMF. The CO_2 _concentration, temperature and humidity of the sham-exposed control samples were similar to EMF-exposed samples. The value of the alternating magnetic field was measured continuously using an EFA-2 field analyzer with a 3 cm diameter probe.

**Real-time PCR for analyzing TERT gene expression. **The ADSCs (from passage four) were cultured at a concentration of 30 × 10^4^ cells per well in 6-well plates for 21 days consist of four groups including group I (control without ZnSO_4_ treatment and EMF exposure), group II (50 Hz, 20 mT EMF exposure without ZnSO_4_ treatment), group III (0.14 µg mL^-1 ^ZnSO_4_ treatment without EMF exposure) and group ІV (50 Hz, 20 mT EMF exposure with 0.14 µg mL^-1 ^ZnSO_4_ treatment). Exposure time duration for EMF-exposed groups was 30 min per day for 21 days. After termination of EMF exposure at the end of 21^th^ day, total RNA was isolated from each group of cells using TRIzol reagent (Invitrogen Life Technologies, Burlington, Canada). The cDNA synthesis was carried out using RevertAid^TM^ first strand cDNA synthesis kit (K1622; Fermentas Life Science, Frankfurt, Germany). The resulting cDNA was stored frozen at –20 ˚C until assayed by real-time PCR. All PCR reactions were performed using Corbett Rotor-Gene (model 6000 HRM; Corbett Research, Sydney, Australia) in a total volume of 20 µL containing 10 µL Power SYBR Green master mix (2x) (TaKaRa Ex Taq HS; Takara Bio, Shiga, Japan), 0.50 µM of each antisense and sense primer as follows: TERT (NM_053423.1): 5'-CAAAAGCCTTTCTCAGCACC-3' (forward), 5'-CTTAATTG AGGTCCGTCCGT-3' (reverse). β-actin (NM_031144): 5'-GCCAACACAGTGCTGTCT-3' (forward), 5'-AGGAGCAATGA TCTTGATCTT-3' (reverse), 1 µL cDNA (30 ng μL^-1^) and 8 µL H_2_O. The PCR amplifications were done in glass capillary tubes. Both β-actin and TERT amplification were done in triplicate for each sample. The thermal cycling conditions were initial denaturation step for 5 min at 95 ˚C, followed by 40 cycles, each denaturation at 95 ˚C for 30 sec, annealing at 59 ˚C for 30 sec and extension at 72 ˚C for 30 sec for both TERT and β-actin.^27^ Cycling threshold (CT) values and the number of β-actin and TERT transcripts in samples were analyzed using Rotor-Gene 6000 software (version 1.7; Corbett Life Science). The CT values were calculated in relation to β-actin CT values by 2^-ΔΔCT^ method.^34^^-^^36^

**Senescence-associated** **β-galactosidase** **(SA-β-gal) activity assay. **To determine the percentage of senescent cells in the presence of ELF-EMF and ZnSO_4_, SA-β-gal staining was used. The assay is based on cytochemical staining for β-galactosidase activity at pH 6. The ADSCs from passages 3, 5, 7 and 9 were plated (2 × 10^3^ cells per cm^2^) in 24-well plates for 21 days consist of four groups including group I (control without ZnSO_4_ and ELF-EMF exposure); group II (ELF-EMF-exposure without ZnSO_4_); group III (ZnSO_4_ treatment without ELF-EMF exposure) and group ІV (ELF-EMF exposure with ZnSO_4_). After termination of EMF exposure at the end of 21^th^ day, cells were washed with PBS and fixed for 5 min with the fixation solution containing formaldehyde and glutaraldehyde. The cells were then, washed twice with PBS, incubated with freshly staining solution containing 40 mM citric acid/Na phosphate buffer, 5 mM potassium ferricyanide, 5 mM potassium ferrocyanide, 150 mM sodium chloride, 2 mM magnesium chloride and 1 mg mL^-1 ^of 5-bromo-4-chloro-3-indolyl-D-β-galactosidase (X-gal) in distilled water over-night (12 to 16 hr) at 37 ^°^C without CO_2_. After incubation, the cells were washed twice with PBS and once with methanol and the dishes were allowed to air dry. A minimum of 100 cells was counted by light microscopy in 10 random fields to determine the percentage of SA-β-gal-positive cells which were appeared as blue-stained cells.^31^^,^^37^

**Statistical Analysis. **The results were analyzed using the software program Graph Pad Prism (version 6.01; Graph Pad Prism, Chicago, USA). We used one-way and two-way ANOVA followed by Dunnett’s post hoc test to determine the significant difference among groups. Statistical significance was determined at *p *< 0.05. All experimental procedures were repeated for three times. 

## Results

**Fig. 1:**
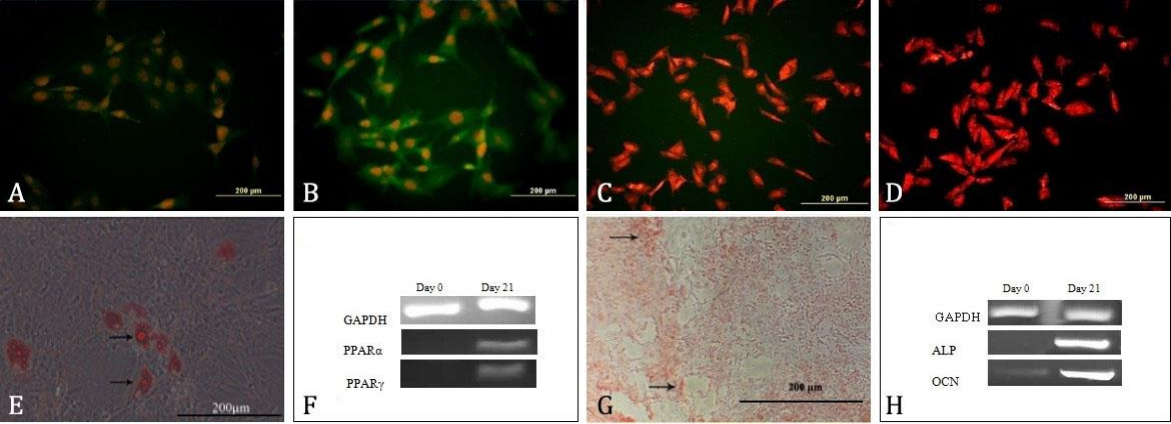
Identification of rat adipose tissue-derived MSCs. Fluorescence microscopy analysis for expression of cell markers of MSCs. A) CD 73, B) CD 90, C) CD 45 and D) CD 56. Nuclei were labeled with PI (orange). E) Adipogenic differentiation. Arrows show lipid vacuoles generated after adipose differentiation. F) Expression of fat-specific genes (PPARα and PPARγ). G) Osteogenic differentiation and cell aggregates (stained with alizarin red staining). Arrows show some of the mineralized cell aggregates (bar = 200μm). H) The RT-PCR analysis and detection of two bone specific genes including alkaline phosphatase (ALP) and osteocalcin (OCN) after osteogenic differentiation

**Phenotypical characterization of ADSCs. **The ADSCs had the capacity to adhere to plastic flasks in culture and morphologically, they appeared as spindle-shaped cells both as scattered individuals and in small colonies. After three passages, red blood cells were seldom seen by microscopy. Immunocytochemical images revealed that cultured cells were consistently positive for CD73 and CD90 and negative for CD45 and CD56 (Fig. 1. A to D). The ADSCs were able to differentiate towards the adipogenic and osteogenic lineages.

**Adipogenic differentiation of ADSCs. **Positive adipogenic differentiation was determined by Oil Red-O staining and molecular analysis. Treated ADSCs with adipogenic differentiation media were stained positive with Oil Red-O staining (Fig. 1E). The expression of peroxisome proliferator-activated receptor-gamma (PPAR-α) and PPAR-γ as adipocyte-specific genes were detected by RT-PCR analyses. These results confirmed that our isolated cells were MSCs.

**Osteogenic differentiation of ADSCs. **The osteogenic differentiation was evident in alizarin red staining. After staining, redness of the nodules indicated the presence of mineralized compartments as a result of the osteogenic treatment (Fig. 1F). The RT-PCR analysis confirmed the expression of bone-specific genes including ALP and OCN in the treated cells.

**Fig.2:**
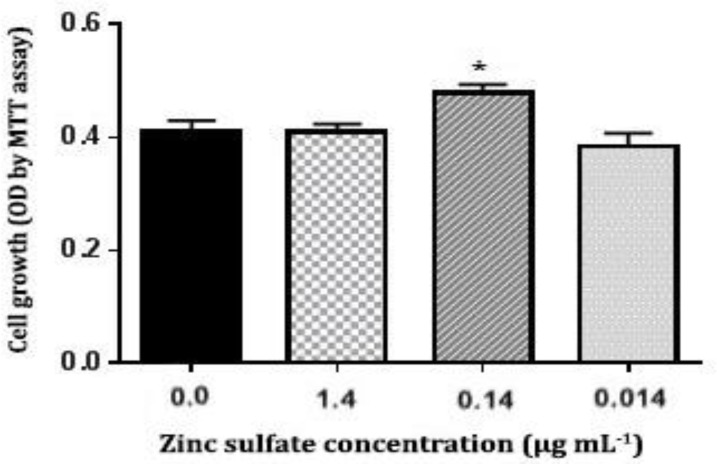
Rat adipose tissue-derived MSCs viability analysis in the presence of different concentrations (1.40, 0.14 and 0.014 µg mL^-1^) of ZnSO_4_ for 21 days by a MTT assay. * indicates significant differences among the groups as *p *< 0.05

**Determination of ZnSO**_4_ **effect on cell proliferation by MTT assay.** As shown in Figure 2, ZnSO_4_ had no significant effect on ADSCs proliferation at concentrations of 1.40 and 0.014 µg mL^-1^, but at concentration of 0.14 µg mL^-1 ^significant proliferative effect was seen (*p *< 0.05). We used 0.14 µg mL^-1 ^ZnSO_4_ in a complete culture medium to treat the cells. 

**Fig. 3:**
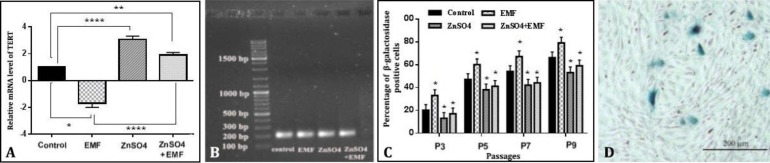
Effect of 0.14 µg mL^-1 ^ZnSO_4_ and 50 Hz, 20 mT ELF-EMF on the mRNA expression level of TERT, analyzed by real-time PCR and also normalized against β-actin. Compared with the ELF-EMF-exposed group in the absence of ZnSO_4_, the mRNA expression level of TERT was significantly higher in cultured cells under ELF-EMF in the presence of 0.14 µg mL^-1 ^ZnSO_4_; B) Detection of TERT gene expression in four groups (control, EMF-exposed, ZnSO_4_-exposed and EMF + ZnSO_4_-exposed) on agarose gel. * indicates significant differences among the groups as *p *< 0.05; while ** *p *< 0.01) and **** p< 0.0001); C) Effect of 0.14 µg mL^-1 ^ZnSO_4_ and 50 Hz, 20 mT ELF-EMF on the β-galactosidase positive cell percentage. Senescent cells were first observed at passage three and their number was increased with passages. Compared with the ELF-EMF-exposed group in the absence of ZnSO_4_, the percentages of β-galactosidase positive cell were significantly lower in cultured cells under ELF-EMF in the presence of 0.14 µg mL^-1 ^ZnSO_4_; D) Senescence related β-galactosidase positive rat adipose tissue-derived MSCs (bar = 200 μm). * indicates significant differences among the groups as *p *< 0.05.

**The effect of ZnSO**_4_ **on TERT expression under 50 Hz, 20 mT EMF. **Real-time PCR for detection of TERT gene expression in ADSCs was carried out after exposure to an electromagnetic field of 50 Hz, 20 mT for 30 min per day for 21 days in the presence and absence of 0.14 µg mL^-1^ ZnSO_4_. The results showed that in groups II, III and ІV (50 Hz, 20 mT EMF exposure with 0.14 µg mL^-1^ ZnSO_4_), TERT mRNA expression decreases (1.70 fold, *p* < 0.05), increases (3.70 fold, *p* < 0.0001) and increases (1.90 fold, *p* < 0.01) as compared with group I, respectively. Meanwhile, in group ІV TERT mRNA expression remarkably increased (4.60 fold) as compared with group II, (*p* < 0.0001; Fig. 3. A and B). 

**Effect of ZnSO**_4_ **on senescent cells under 50 Hz/20 mT EMF. **The SA-β-gal activity assay for detection of senescent cells was carried out after exposure to an EMF of 50 Hz, 20 mT for 30 min per day for 21 days in the presence and absence of 0.14 µg mL^-1 ^ZnSO_4_. The results showed that in group II, percentage of β-galactosidase positive cells was increased as compared with group I (*p *< 0.05). Meanwhile, percentage of β-galactosidase positive cells in group III and group ІV was decreased as compared with group I (*p *< 0.05) (Fig. 3 C and D).

## Discussion

Due to increased use of ELF-EMFs for domestic and industrial appliances in the last few years, different *in vivo* and *in vitro* studies have been conducted on the influence of these fields on various biological functions such as tumor growth, neurodegenerative diseases, free radical production, gene expression, growth, aging and differentiation potential of MSCs.^29^ Since the MSCs are attractive candidates for cell-based tissue regeneration, finding EMFs effects on MSCs is important for researchers.^38^^,^^39^ Aging of MSCs is one of the conditions that may be caused by EMFs. Aging is an inherently complex process that is manifested within an organism at molecular and cellular levels. While the fundamental mechanisms are still poorly understood, telomere shortening and reduction of TERT expression are one of the cellular markers of aging and/or cell death.^40^ Molecular control of stem cell aging is highly regulated by two different, telomerase-independent and telomerase-dependent pathways.^16^ Epigenetic events play a role in the telomerase-independent senescence pathway. Therefore, finding ways to reduce the replicative senescence is definitely attractive in cell transplantation approaches. The results of EMFs studies are different because of the difference in the magnetic field intensity and frequency and duration of exposure.^5^ Reportedly, REAC acts on a gene and protein expression program of both telomerase-independent and telomerase-dependent patterns to optimize stem cell ability to cope with senescence progression.^15^ It seems that gene expression regulation may be directly involved in differentiation potential and aging in stem cells. It has been demonstrated that TERT over-expression can prevent the human MSCs senescence and the cells showed significantly higher and unlimited proliferation capacities.^41^ It was found that 50 Hz, 0.50 and 1 mT magnetic fields can promote survival and proliferation of the human ADSCs.^42^ There is no substantial evidence for the effects of ELF-EMF on TERT expression. In this regard, an understanding of the TERT expression underlying the regulation of telomerase activity might allow the modulation of telomerase expression and thus, cell lifespan. At present, finding ways to reduce the reduction of TERT expression and cellular senescence caused by ELF-EMF is definitely attractive. In the present study, 0.14 µg mL^-1 ^was used as the final concentration of ZnSO_4_. To determine a suitable range for ZnSO_4 _under this study experimental conditions, MTT assay was used to evaluate cell viability. In this study, it was discovered that ZnSO_4_ has no impact on the viability of cells below 0.14 µg mL^-1 ^and above 0.14 µg mL^-1^. The previously described exposure condition was used in this study.^4^ Real-time PCR was used to explore the mechanism of ELF-EMF in the presence of ZnSO_4_ on ADSCs aging at the molecular level and the mRNA expression of TERT was detected. The results of this study showed that in cultured cells under EMF in the presence of 0.14 µg mL^-1 ^ZnSO_4_ (group IV), TERT mRNA expression remarkably increased (4.60 fold) when compared with EMF-exposed group (group II). Previous reports provided the first evidence that metals, especially Zn^+2^ can modulate telomerase in cancer cells by inducing an enhancement of its activity.^18^^,^^43^ On the contrary, it has been reported that zinc phthalocyanine (ZnPc) has the ability to inhibit telomerase activity.^44^ In this study, β-galactosidase staining method was used as a common method to study cells senescence* in vitro*, in order to examine the percentages of the senescent ADSCs in our studied culture condition.^45^ According to our observations, ß-galactosidase positive cells were first observed at passage three and their number increased as the passage number increases. The results of the present study showed that senescence related β-galactosidase positive cells at passages 3, 5, 7 and 9, under EMF in the presence of 0.14 µg mL^-1 ^ZnSO_4_ (group IV) decreased significantly when compared with the EMF-exposed group (group II). The effects of ZnSO_4 _on TERT gene expression and aging of ADSCs under ELF-EMF are yet to been investigated, but the role of Zn^+2^ in growth regulation was evaluated.^46^ However, the findings of this study are in agreement with the hypothesis that 0.14 µg mL^-1 ^ZnSO_4_ as an antioxidant can prevent the aging of ADSCs under 50 Hz, 20 ELF-EMF by increasing TERT gene expression and ADSCs aging reduction. Although the underlying mechanism remains unclear, but few studies have been published confirming the effectivness of zinc on telomeres. Reportedly, octa-cationic zinc phthalocyanine (ZnPc) can induce intra-molecular G-quadruplex telomeric structure transition from the antiparallel to parallel form.^44^ It has been shown that 80 µm L^-1^ ZnSO_4_ helps to maintain and shorten the telomere length of hepatocytes L-02 and hepatoma cells SMMC-7721.^47^ It was found that the molecular mechanism might be related to inhibition of telomerase activity in SMMC-7721 cells. It might also be the result of p53 protein activation, a zinc-binding transcription factor that responses to multiple forms of stress and controls proliferation, survival, DNA repair and cells differentiation. Therefore, supplementation of zinc could affect the transcription of DNA and telomere length by activation of p53 in hepatoma cells.^48^ According to the aforementioned reports, in the present study, it was suggested that ADSCS aging prevention in the presence of ZnSO_4_ may be due to changes in TERT gene expression, telomerase activity, telomere structure and telomere length.

In conclusion, the findings of this study showed that the TERT gene expression significantly increased while the percentage of senescent cells decreased under ELF-EMF of 50 Hz, 20 mT in ZnSO_4_-treated ADSCs when compared to untreated cells. The results suggested that 50 Hz, 20 mT magnetic field could affect the senescence and cellular aging due to TERT gene expression; although, the detailed mechanisms are still unclear. More studies are recommended on the effects of magnetic fields on telomere length and telomerase activity of stem cells. 
